# Use of the directional atherectomy for the treatment of femoro-popliteal lesions in patients with critical lower limb ischemia

**Published:** 2016-11-01

**Authors:** Umberto Marcello Bracale, Gaetano Vitale, Guido Bajardi, Donatella Narese, Ettore Dinoto, Anna Maria Giribono, Doriana Ferrara, Luca del Guercio, Massimo Midiri, Felice Pecoraro

**Affiliations:** 1 Department of Vascular and Endovascular Surgery, University Federico II of Naples, Naples, Italy; 2 Vascular Surgery Unit, University of Palermo, Palermo, Italy; 3 Department of Radiology, DIBIMED, University of Palermo, Palermo, Italy

**Keywords:** Turbohawk device, critical limb ischemia, atherectomy, endovascular treatment

## Abstract

Femoro-popliteal PTA for the treatment of critical limb ischemia is frequently associated with unsatisfactory procedural success rates while directional atherectomy (DCA) has improved success rate since claudicant patients undergoing percutaneous treatment of femoro-popliteal obstructive disease. The aim of this prospective study is to evaluate the safety, efficacy and procedural success of DCA, at one year, in the percutaneous treatment of femoro-popliteal obstructive disease in patients with critical limb ischemia.

**Methods:**

From March 2012 to March 2013 18 consecutive patients with critical limb ischemia were treated with DCA (Turbohawk/Covidien-ev3 Endovascular Inc., North Plymouth, Minnesota, USA) for the treatment of femoro-popliteal obstructive disease. Patients were evaluated at 12 months.

**Results:**

Technical and procedural success was achieved in every patient. No in-hospital major adverse cardiovascular events occurred. Primary endpoint: freedom from any amputation was obtained in all patients. Secondary endpoints: clinical (Rutherford class improvement) and hemodynamic success (Ankle-brachial index improvement) was achieved in all patients.

**Conclusion:**

The use of DCA for the treatment of femoro-popliteal obstructive disease is a safe and effective therapeutic strategy for patients with critical limb ischemia. The data included in our study should be considered hypothesis-generating in order to design of a randomized trial comparison with conventional PTA.

## I. INTRODUCTION

In developed countries of the western hemisphere, atherosclerotic critical limb ischemia (CLI) has an estimated incidence rate of at least 500 new cases per million habitants per year [1]. CLI revascularization is the mainstay of modern vascular therapy. Among non-revascularized patients, 50% will undergo a major amputation one year after CLI onset. Perioperative mortality for above and below-the-knee amputation is 5–10% and 15–20%, respectively [2]. Percutaneous transluminal angioplasty (PTA) is the first treatment option in CLI and femoro-politeal obstructive disease patients, particularly those with short arterial lesions. However such procedures often result in low acute success rates due to the difficulty of dilating fibrocalcific atherosclerotic plaque and, as a consequence, frequently require stent deployment [3,4]. Directional atherectomy (DCA) in claudicant patients has improved acute success rates by debulking the fibro-calcific portion of the atherosclerotic plaque thereby achieving proper lumen dilation and avoiding stent deployment (Rutherford Clinical Categories 2–3) [5].

The aim of this prospective study is to evaluate the safety and efficacy, at one year, of the directional atherectomy procedure and assess its impact on the procedural success of endovascular treatment for femoro-popliteal obstructive disease in patients with critical limb ischemia.

## II. METHODS

### Patient population

From March 2012 to March 2013, 18 consecutive patients with critical limb ischemia underwent DCA (TurboHawkTM plaque excision system, Covidien/ev3, Plymouth, MN) at two university hospital for treatment of femoro-popliteal obstructive disease. Patients were treated as standard in our practice and included in a prospective registry. Both the hospitals Ethics committee approved follow-up protocol.

Inclusion criteria were:

- clinical signs of critical limb ischemia: categories 4–6 according to the Rutherford classification
- superficial femoral artery (SFA) stenosis >50% or popliteal artery stenosis (PA) >50%
- lesion length < 30 cmat 1, 3, 6 and 12 months to evaluate occlusion or restenosis [5].
- reference vessel diameter > 4 mm
- Duplex sonography (DUS) preoperative evidence of fibro/calcific lesion

All patients were preoperatively evaluated with clinical examination, ABI measurement, Duplex scan and angiography, in order to define Rutherford Clinical Category (RCC), ecographic type of lesion and TASC category.

Exclusion criteria were:

- clinical sign of acute limb ischemia
- in-stent restenosis
- concomitant aneurysmatic disease
- subintimal recanalization

### Concomitant therapy

All patients were given acetylsalicylic acid (ASA) (100 mg/day) and were instructed to take clopidogrel (75 mg/day) for at least 7 days. Alternatively, patients received clopidogrel preload (300 mg) 24 hours before procedure. Post procedure, thyenopiridines were administered and continued for 30 days and ASA was prescribed for life. For anticoagulation, 70–100 UI/Kg of un-fractioned heparin (UFH) was administered intravenously at the start of the procedure in accordance with our previously-described endovascular protocol [6]. Likewise, prevention of contrast-induced nephropathy (CIN) was careful monitored [7].

### Procedure

**Fig. 1:**
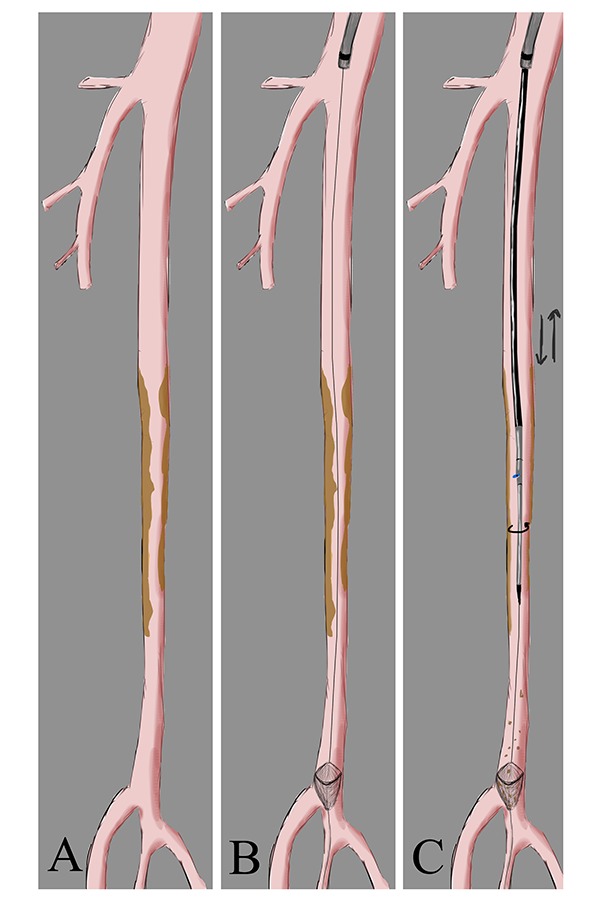
Illustration of the Turbohawk™ device. **A**. Multiple preocclusive SFA stenosis. **B**. Deployment of distal protection device. **C**. Plaque excision with Turbohawk™ (description of the technique in the text).

**Fig. 2:**
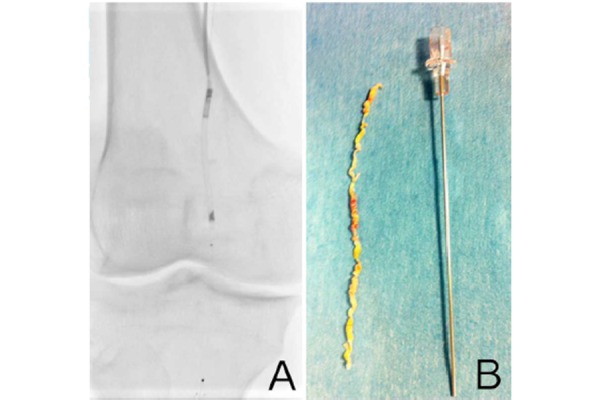
**A.** intraoperative image showing Turbohawk™ device and distal protection filter SpiderFX™. **B.** Long trimmed plaque.

**Fig. 3:**
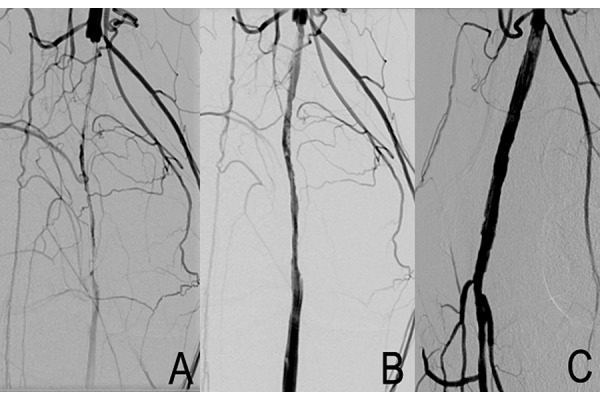
Intraoperative angiogram. **A**. Superficial femoral and popliteal artery occlusion. **B.** Femoro-popliteal pre-dilatation. **C.** Postprocedural result after plaque excision with Turbohawk.

All procedures were performed under local anaesthesia with the patient in a supine position. Vascular access was percutaneously achieved via the ipsilateral common femoral artery. In two cases a surgical cut-down was necessary, with a purse string suture positioning on the artery before the puncture; one due to the patient’s obesity (BMI 32 Kg/m2) and the other to treat an SFA ostial lesion. An 8 Fr sheath was advanced antegradely in all 18 patients’ SFA in order to achieve adequate support. Once diagnostic angiography was completed, a 0.014″ guidewire was advanced into the distal popliteal artery. A filter for embolic protection (Spider FX™ embolic protection device (EPD), Covidien, Plymouth, MN) was positioned distal to the stenosis. Filter size was chosen according to landing artery diameter. DCA was performed using the Turbohawk™ device; a monorail exchange system running over a 0.014″ guidewire which was chosen out of the 7 diameters and catheter lengths available to treat the femoral, popliteal and below the knee vessels. The device consists of a plaque blade cutter and reservoir at the tip, which stores the trimmed plaque. Under fluoroscopic guidance we advanced the device across the lesions at a speed of 1–2 mm per second with the cutting blade in constant orientation against the eccentric plaque, thereby avoiding circular movements. The cutting sequence was repeated as necessary in order to excise the largest amount of plaque possible (Fig. 1) (Fig. 2) (Fig. 3). Self-expanding nitinol stent implantation was permitted for bailout stenting (residual stenosis > 30% or flow limiting dissections). Lesion pre-dilation was left at operator’s discretion.

### Post-procedural patient management

Access site haemostasis was achieved by manual compression in all percutaneous cases. Surgical access was closed gently pulling the purse string suture, after sheath removing, skin sutures completed the procedure. No vascular closure devices were used in this study.

### Patient Follow-up

Patients were evaluated at hospital discharge, at 30 days, and at 3, 6 and 12 months post procedure. Clinical follow-up was performed by clinical examination, ABI measurement and duplex ultrasonography scan. Repeat angiography was performed when proximal flow velocity ratio (PVR) was between 2.4 to 5.0 (intermediate re-stenosis) and patient had clinical symptoms or PVR greater than 5.0 (severe re-stenosis), irregardless of clinical symptoms and in cases of vessel occlusion [8].

### Definitions

Technical success was defined as the ability to successfully perform DCA with a residual stenosis of <30%. Procedural success was defined as technical success without the occurrence of any major in-hospital adverse cardiovascular events (MACE).

Amputations distal to the metatarsal region were considered as limb salvage.

Primary endpoint was limb salvage at one year.

Secondary endpoints included:

- primary patency at one year.
- clinical success as defined by >1 category improvement in the RCC from baseline (or 2 categories if pre-existing tissue loss) at one year.
- hemodynamic success as defined by a 0.1 improvement in the ABI during the period from baseline to 30 days post-procedure and no deterioration > 0.15 from the maximum early post procedure level at one year.
- patency of below-the-knee artery as defined by freedom from obstructive lesions determining angiographic stenosis > 70%.

### Statistics

Nominal and categorical variables were presented as contingency tables with frequencies and percentages. Continuous variables were reported as the mean with standard deviation or median and interquartile ranges. Variables were compared by t test for normally distributed values (probability value <0.05 was considered statistically significant).

## III. RESULTS

Out of the 18 patients included in the study, 12 (67%) were male. Demographic and clinical data are reported in Table I. Sixteen (88.9%) of the patients presented multiple SFA stenoses. In the remaining two cases a femoropopliteal artery occlusion was noted (11.1%). Mean length lesion was 12.3 cm. In 14 cases length was > 10 cm. In 16 (88.9%) patients’ lesions were classified as TASC C. In the remaining two cases (11.1%) as TASC B. Lesion predilatation was necessary in seven (38.9%) cases, five of which were due to the presence of pre-occlusive stenosis in the patients while the other two had total vessel occlusion.

**Fig. 4:**
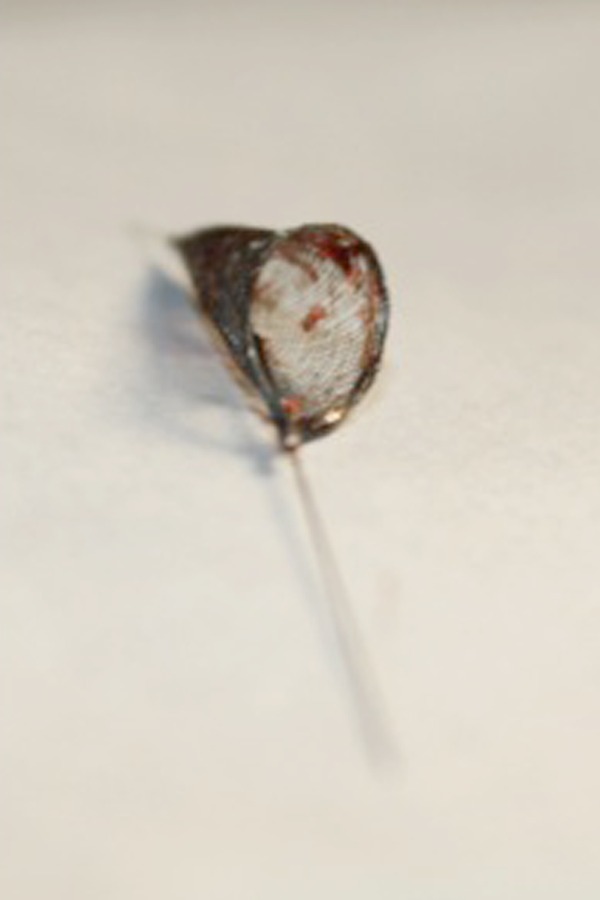
Macroscopic embolic material after distal embolic protection filter removal.

DCA was performed on all 18 patients with an ensuing technical success achieved in all cases (100%). No vessel perforation occurred. In no cases was bailout stent implantation necessary. Six (33.3%) diabetic patients with distal ulcers required additional tibial PTA in order to ensure direct blood flow to the foot. Procedural characteristics are summarized in Table II. Embolic material was evident at macroscopic inspection within the positioned filter in all of the cases (Fig. 4) thus demonstrating embolization of atherosclerotic debris. In only one case (5.6%) was the debris amount large enough to determine filter plugging. A normal antegrade flow was restored following filter recapture. No MACE occurred in hospital and, consequently, procedural success was also achieved in 100 % of the cases. No deaths or major amputations occurred during follow-up. Limb salvage was achieved in all patients while two cases required minor toe amputation.

One-year duplex scan assessment revealed no recurrent stenosis or occlusions in 15 of the patients, giving a primary patency of 83.3%. In one of patients an SFA reocclusion occurred at 6 months however no signs of CLI recurrence were observed (Rutherford 3). Two other patients received femoro-popliteal below-the-knee bypass determining a secondary patency rate of 94.4% at 12 months.

Preoperatively mean ABI was 0.35 ± 0.2. At one year ABI increased significantly to 0.60 ± 0.32 (P < .001). All patients had improved clinical status according to Rutherford classification (Baseline and follow up data to be inserted). Mean procedure time was 99 minutes (range 80–122; SD: 30) and mean fluoroscopy time was 29 minutes (range: 21–35; SD: 7.5).

## IV. DISCUSSION

Nowadays the use of endovascular therapies as primary treatment in patients with CLI and femoro-popliteal lesions is on the rise. However the success of interventional endovascular therapy becomes hampered in the presence of calcific disease [9] while heavy calcification is a strong predictor for bailout stenting [10]. DCA improves acute success in claudicant patients by debulking the fibrocalcific portion of atherosclerotic plaque and achieving proper lumen dilation thereby avoiding stent deployment [5]. However initial results in patients with CLI and severely calcified femoro-popliteal vessels were unsatisfactory leading to stagnation in the clinical use of atherectomy [11]. Recent developments in atherectomy technology have renewed enthusiasm for the DCA technique. The TurboHawkTM plaque excision system, (Covidien/ev3, Plymouth, MN) was approved for clinical use by the FDA back in 2011 and represents an evolution in atherectomy therapy when compared to the widely employed and well-studied SilverHawkTM debulking device (Covidien/ev3, Plymouth, MN) system due to its thinner hub-based rotating blade situated behind a tapered, debris-collecting nose cone. The ‘TALON Registry’ described the first multicenter experience with the SilverHawk™ device on 601 patients with 1258 symptomatic lower extremity atherosclerotic lesions (748 limbs) and a reported technical success rate of 94.7%. At one year primary patency was reported in 81% of the cases. Out of the 1258 total, 245 were patients with CLI [12].

In a recently published single-center prospective study, the use of SilverHawk to treat calcified femoro-popliteal lesions achieved a very good peri-procedural success rate (92%) with minimal need for bailout stenting (8%) [13]. In the DEFINITIVE LE study 25% of the patients enrolled had CLI and bailout stenting was necessary in 4.1% of the cases, despite the presence of moderately or severely calcific lesions [14].

The use of DCA can be complicated by the occurrence of distal embolization. The value of using embolic protection with atherectomy is controversial and varies according to lesion characteristics and disease severity (i.e., CLI or single vessel run-off) [15]. As well, calcification has been shown to increase the risk of clinically significant embolism [16]. In the DEFINITIVE LE study, debris was present in 88.4% of distally placed filters upon recovery. Only three angiographically evident embolic events (2.3%) were reported, none of which resulted in clinical sequelae [17]. This embolization rate was comparable to stenting and PTA [18], notwithstanding the challenging calcified lesion population in the study.

In our experience, attributed to a combined approach of distally protected DCA, procedural success was achieved in all the patients, resulting in a primary patency rate of 94.4% at 12 months. Distal EPDs were placed in all patients and in 100% of the cases macroscopic atherosclerotic material was evident at filter removal. In one case the high amount of debris determined a ‘noflow’ image during intraoperative angiography. These findings demonstrate that embolization continues to be a concern for atherectomy devices suggesting the routinely use of distal EPDs with associated additional cost and procedural overload. Recently there is reasonable attention on the assumption of using DCA before drug-eluting balloon angioplasty considering that DCA removes plaque that may hamper the passage of the drugs to the vascular media. Preliminary experience in a small cohort of patients (60% were claudicants) demonstrated safety and efficacy of this combined treatment [19]. The vast majority of published data available on the use of DCA for femoro-popliteal intervention is based on the employment of the Silverhawk™ device. As was the case for Silverhawk™ when it was first employed and approved by FDA in 2007, an adequate learning curve is necessary to confidently master the use of the Turbohawk™ device [18,20,21]. We found that its main advantage is that it allows for more consistent plaque removal due to its technologically advanced design; an essential function given the large atherosclerotic burden of the CLI patients.

## V. CONCLUSIONS

Our prospective study of 18 patients at a 12-month follow-up has demonstrated that:

- DCA can be safely performed to treat femoro-popliteal obstructive disease in patients with CLI.
- The use of DCA is associated with good clinical outcome and low recurrence rates.
- The finding of macroscopic plaque debris in all patients is strong supporting evidence for the systematic use of the distal embolic protection device.

Endovascular atherectomy with distal protection should be considered a treatment option for complex calcified disease in the lower extremities. Limitations of this study include lack of a control arm, a relatively small number of patients and long-term follow-up.

This study was designed to include a specific cohort of patients and selection criteria could be considered in the design of an eventual, larger study.

## Figures and Tables

**TABLE I:** DEMOGRAPHIC AND CLINICAL DATA

	N= 18 (%)
Male	12 (66.7)
Age, years (Median/IQ)	68.1 (56–83)
Hypertension (%/N)	16 (89)
Hypercholesterolemia (%/N)	12 (67)
Diabetes Mellitus (%/N)	14 (78)
Smoking history (%/N)	12 (67)
Chronic renal disease (%/N)	2 (11)
Rutherford 4 (%/N)	2 (11)
Rutherford 5 (%/N)	6 (33)
Rutherford 6 (%/N)	10 (56)
CVD (%/N)	4 (22)

**TABLE II:** PROCEDURAL CHARACTERISTICS

	N = 18 (%)
Pre-dilation	7 (38.9)
Distal protection	18(100)
Reference Vessel Diameter (mm) (Mean ± SD)	4.1 ± 1.2
Lesion Length (mm) (Mean ± SD)	123 ± 55
Post-dilation	3 (16.7)
Bailout stenting (%)	0 (0)
Procedural success	18 (100)
